# Effectiveness of a muticomponent workout program integrated in an evidence based multimodal program in hyperfrail elderly patients: POWERAGING randomized clinical trial protocol

**DOI:** 10.1186/s12877-019-1188-x

**Published:** 2019-06-21

**Authors:** Manuel González-Sánchez, Antonio Ignacio Cuesta-Vargas, María del Mar Rodríguez González, Elvira Díaz Caro, Germán Ortega Núñez, Alejandro Galán-Mercant, Juan José Bedoya Belmonte

**Affiliations:** 1 Department of Physiotherapy, Faculty of Health Sciences, Instituto de Investigación de Biomedicina de Malaga (IBIMA), Universidad de Malaga, Málaga, Spain; 2 School of Clinical Science, Faculty of Health, Queensland University of Technology, QLD, Kelvin Grove, Australia; 3 Servicio Andaluz de Salud, Distrito Sanitario Málaga. CS. Tiro Pichón, Instituto de Investigación de Biomedicina de Malaga (IBIMA), Malaga, Spain; 4 Department of Health Sciences, University of Jaen, Jaen, Spain; 5 MOVE-IT Research group and Department of Nursing and Physiotherapy, Faculty of Nursing and Physiotherapy University of Cádiz, Cádiz, Spain; 6 Biomedical Research and Innovation Institute of Cádiz (INiBICA) Research Unit, Puerta del Mar University Hospital University of Cádiz, Cádiz, Spain

**Keywords:** Frailty, Physical activity, Physical fitness, Falls, Diet, Elderly

## Abstract

**Background:**

Short-term and mid-term comparison of the efficacy of a multimodal program that incorporates a therapeutic workout program, medication review, diet adjustment and health education, in comparison to the standard medical practice in the improvement of the neuromuscular and physiological condition. Furthermore, it is intended to analyse the maintenance of these effects in a long-term follow-up (12 months) from the onset of the intervention.

**Methods:**

A randomized clinical trial of elderly frail patients drawn from the Clinical Management Unit “Tiro de Pichón”, Health District of Malaga, will be included in the study (after meeting the inclusion / exclusion criteria) will be randomized in two groups: a control group that will undergo an intervention consistent of medication review + diet adjustment + health education (regular workout recommendations within a complete advice on healthy lifestyles) and an experimental group whose intervention will consist of a multimodal treatment: therapeutic workout program+ medication review+ diet adjustment + health education. The sociodemographic, clinical and tracing variables will be reflected at the beginning of the study. In addition, the follow-up variables will be gathered at the second and sixth months after the beginning of the treatment and at the third and sixth months after the treatment (follow-up). The follow-up variables that will be measured are: body mass index, general health condition, fatigue, frailty, motor control, attention- concentration- memory, motor memory, spatial orientation, grip strength, balance (static, semi-dynamic), gait speed and metabolomics.

A descriptive analysis of the sociodemographic variables of the participants will be conducted. One-Factor ANOVA will be used for the Within-Subject analysis and as for the Between-Subject analysis, the outcome variables between both the groups in each moment of the data collection will be compared.

**Discussion:**

A multimodal program that incorporates a therapeutic workout program, medication review, diet adjustment and health education may be effective treatment to reduce the functional decline in elderly. The results of the study will provide information on the possible strengths and benefits in multimodal program in elderly.

**Trial registration:**

ClinicalTrials.gov NCT02772952 registered May 2017.

## Background

In the year 1990, the WHO defines the elderly population as people aged 65 or above [1]. In the recent years, a new paradigm of aging has emerged that has been addressed as: healthy, good aging, competent, successful or active [2]. This concept has evolved, back from the definition of healthy aging given by the WHO in 1990 (health centralized), towards a much more integrated model, such as the one of active aging [3]. Aging is a deleterious, progressive, intrinsic and universal process that eventually occurs in all living entities as a result of the interaction of a living being’s genetics and its environment [4]. The ways of aging are neither determined nor prefixed; and although we can’t ignore that there are genetic variables that play an important role in aging, the individual is also responsible for a, more or less, satisfactory and free from dependence aging, with its actions and behaviour throughout its life [2], hence making it possible to discuss three basic forms of aging: usual, pathological and optimal aging [2].

According to the recent estimations, the world population above 65 years old were 528 million in the year 2010, and will be more than 605 million in the year 2015. The United Nations forecast for Spain in the year 2015, for the population above 65 years, is estimated in more than 8 million of subjects [5], entailing more than 18% of the Spanish population (CNE, Centro Nacional de Epidemiología). The Ministry of Health and Consumer Affairs claims that life expectancy draws an ascendant progression in most of the developed countries; nevertheless, that doesn’t necessarily imply that the increased number of years in this indicator would be years in a good health condition. In most of the cases, the elderly population are affected by diseases and health issues that result in quality of life deterioration, even if they don’t occur in immediate death [6].

Aging has been traditionally treated as rather “pathological” [7]. Nonetheless, this vision is being replaced by another one that defends that, although there are certain diseases that indeed appear more frequently in the old age than in other stages of life, this doesn’t mean that both of these aspects are intrinsically associated [7]. In fact, there is enough empirical evidence to assert that both health care interventions and lifestyle habits significantly contribute to an improvement of a life expectancy without any impairments [7, 8].

Hence, there is a pressing need of identifying those factors that influence the onset of impairment and dependence in the elderly, both of which notably deplete the quality of life in this age group [7, 8]. It is in this context that the concept of “frailty” appears, a concept that refers to something that can relatively deteriorate with ease [7, 8]. Currently it’s defined as a biological syndrome with a decrease of the reserve and endurance to physical and psychological stress factors that increase the vulnerability to adverse situations [7]. This concept has gained importance since its inception and it has risen to take an important role in the scientific literature on aging. At present, frailty is assumed to be a construct that often underlies health deterioration that occurs in the elderly, there being an emerging agreement that the frailty markers include age related reduced lean body mass, strength, endurance, balance, walking speed and low activity [8].

Moreover, while frailty, impairment and dependence typically coexist, frailty seems to be prior and, thus, likely to be early intervened on [9]. Therefore, it’s crucial to specifically identify the features of the people that confirm the group of “elderly frail” being convinced that this information could help to elaborate targeted interventions to prevent and/or to a more effective intervention in the courses of impairment and dependence. It is here where the starting point of the current investigation work is established.

Frailty commonly has a major impact on the patient’s quality of life, leading to undesirable consequences in the physical capacities, intellectual activity, emotional aspects, personal relations and cognitive issues [10]. Among the most frequent cognitive problems, decreased selective attention, loss of memory, mental confusion, and concentration difficulties, are described [11]. Despite there being a great emphasis to the assessment of the cognitive problems, these shouldn’t be solely interpreted, as the consequences could have a negative reflection on the functional impairment, depressive states, sleep pattern variations, and even social vulnerability [12]. A reliable, inexpensive and valid way to identify the effect that the patient suffers is using self-administered surveys [13]. The results of patient-provided measures are used as an assessment and/or follow-up tool to objectively reflect the patient’s health condition or its functional status at a specific moment. These tools help the physicians to understand the impact of a disease or condition over the patient’s symptoms, performance and skills.

Furthermore, changes and modifications that occur as a result of frailty, are reflected in the body’s metabolic balance [14]. These changes can be collected and analysed through the use of metabolomics (a technique that comprises the development of systematic profiles from the multiple metabolite concentrations and their cellular and systemic fluctuations in response to drugs, diet, lifestyle, environment, stimuli and genetic modulations, in order to distinguish beneficial and adverse effects of such interactions [15]. By systematically measuring biomarkers (metabolites) in the population, profiles can be set among healthy individuals from those with specific diseases [16]. Moreover, metabolomics may provide signs of a metabolic disorder or a lesion with high precision and less cost than genomics, transcriptomics or proteomics [17].

Finally, the evaluation of the physical function is an important component of the geriatric assessment in the elderly [18, 19]. These measurements have shown to have sufficient discriminating sensitivity and have proven the ability to predict the outcomes, such as risk of falls, institutionalization, and death [20, 21]. There are tests based on the strength, walking speed or the transition between the different daily life positions that have proven to have a high sensitivity in the diagnosis of frailty [19, 21]. This set of tests to assess fitness and performance have proven to be reliable and valid for assessing the functional impairment in the elderly that live in nursing homes [20]. Recently, several studies have examined the functional physical function which make up the functional test and the instruments used, together with improvements to the instruments, specifically attaching inertial sensors to the body [22–44].

The specific applications for these elements are already being developed for use in a wide range of situations [44, 45]. Such uses include assessing and quantifying kinematic variables related to functional tasks [24–26, 43], identifying trembling in people suffering from Parkinson’s disease [22] and measuring Cobb angles in X-rays [45]. Another application is the development of an objective method to classify levels of physical activity and an indicator of the degree of functional capacity and quality of life [46] and gait characteristic analysis and identification based on the smartphone’s accelerometer and gyrometer [47].

Therefore, the use of different assessment methods by means of which the elderly are analysed from different perspectives (physical, mental, metabolomics, subjective, etc.), could help to identify the elderly frail, which would enable setting up the intervention protocols intended to reverse theses deficient states or at least, slowing their progression.

In this respect, in order to planning any intervention, it’s important to note that the quality of muscle mass, namely, force production per unit of active muscle mass, has been associated to the functional ability in the elderly population [48]. Muscle quality provides an estimate of the contribution of the neuromuscular factors related to changes in the development of strength, as when the strength is increased while maintaining the same muscle mass, this suggests neural adapting to training [49].

In this sense, a combination of strength and endurance training in the elderly population has been suggested to be the most effective strategy to improve both neuromuscular and cardiorespiratory functions, the preservation of the functional ability and health promotion during old age [50, 51]. However, several studies have proven that in the simultaneous training, the “interference effect” may appear, reducing the strength adaptations when it is compared to only strength training [52], even when the participants of the training protocols are the elderly, [50, 51], being noticed not only in the strength training, but as well in those that are intended to a better cardiorespiratory performance in the elderly population [50, 51]. Thus, along with the amount of aerobic training and intensity, within-session workout sequence could be an important variable in the prescription of simultaneous training [50], as the strength performance just before the endurance training compromises endurance gaining [50].

A decrease in muscle quality has been associated with decreased functional ability in the elderly population [48]. To counteract this process, some studies have proven that quality strength training increases muscle mass in the elderly [49–51].

## Methods/design

### Hypothesis

The interaction of various intervention strategies, wherein a supervised workout program, medication review, diet adjustment and health education are included, should achieve further improvements of the neuromuscular and physiological state in the elderly identified as frail, than the standard medical practice.

### Objective

Short-term and mid-term comparison of the efficacy of a multimodal program that incorporates a therapeutic workout program, medication review, diet adjustment and health education, in comparison to the standard medical practice in the improvement of the neuromuscular and physiological condition. Furthermore, it is intended to analyse the maintenance of these effects in a long-term follow-up (12 months) from the onset of the intervention. The workout program consists of a specific part for the development of strength followed by another specific part for endurance training. This sequence has better results than if structured inversely according to previous study [50].

### Design and study patients

The design of the present study is a randomized clinical trial that will be developed in frail elderly people. The present study is a randomized controlled clinical trial conforming to Consolidated Standards of Reporting Trials (CONSORT) guidelines. We refer to hyperfrail to the patients located in the 1/3 of the screening normalized scores, coming from the Clinical Management Unit “Tiro de Pichón”, Health District of Malaga. The inclusion criteria that were established were patients older over 65 years old that, after screening of frailty according to the Short Physical Perfomance Battery (SPPB) series of tests recommended in the Consensus Document by the Ministry of Health, Social Services and Equality on prevention of frailty and falls in the elderly patients, could carry out the program of therapeutic workout [53]. The information and signature of the informed consent are indispensable.

The exclusion criteria established were the presence in the clinical history record of neuromuscular, metabolism, hormonal and/or cardiovascular disorders.

This study will be carried out in accordance with the principles of the Helsinki Declaration to ensure the protection of the rights, safety and welfare of the volunteers that participated in it. Ethical approval of the study will be granted by an Ethical Committee of a public institution [Investigación Provincial de Málaga (Consejería de Salud Servicio Andaluz de Salud, Spain)].

### Outcome measures

Expanded Table 1 with reliability, reference and brief description.

**Table 1:** Study variables list

Variable	Type	Value
Socio-demographic		
Gender	Categorical/Dichotomous	Male/Female
Age	Continuous Quantitative	Numeric. Range
Education level	Categorical/Polychotomous	No education/Basic education/Higher education
Clinical		
Risk of falling record	Categorical/Dichotomous	Yes/No
Causes of falling	Categorical/Polychotomous	Intake of psychotropic drugs, presence of urinary incontinence, cognitive impairment, visual problems
Workout	Categorical/Dichotomous	Yes/No
Obesity	Categorical/Dichotomous	Yes/No
FOLLOW-UP		
Body Mass Index (BMI)	Continuous Quantitative	Numeric. Range
General health condition	Continuous Quantitative	SF-12 V2
Fatigue	Continuous Quantitative	POMS (Profile of Mood States)
Frailty	Continuous Quantitative	Inter-Fraitly Questionnaire
Motor control	Continuous Quantitative	Mini-Mental State Examination
Attention – concentration – memory	Continuous Quantitative	WAIS III – Digit Symbol test.
Motor memory	Continuous Quantitative	10 positions – 10 s
Spatial Orientation	Continuous Quantitative	Path reproduction
Grip Strength	Continuous Quantitative	Hand – Grip test
Balance (static, semi-dynamic)	Continuous Quantitative	Time Get-up and Go (extended version)
Gait speed	Continuous Quantitative	Test: 20 m max. Walking speed
Metabolomics	Continuous Quantitative	Systemic inflammation, glucose and lipid metabolism

### Anthropometry

The measurements will be taken following the anthropometric parameter guidelines of The International Society for the Advancement of Kinanthropometry [54] (ISAK)

- Weight: the subject would be barefooted and in undergarments.
- Height: distance from the vertex to the sole of the foot. The subject would be standing, in anatomical position and with the occipital region, back, buttocks and heels in contact with the measuring rod. The subject will take a deep breath at the time of measurement while keeping the head in the Frankfort plane.

Body Mass Index (BMI) is obtained dividing the weight in Kilograms (Kg) by the square of height in metres. It estimates the amount of fat and lean mass of the body.

### General health condition using the short form SF-12 questionnaire

General health condition was measured using the SF-12 short form questionnaire, adapted from the extended SF-36 version. Both physical and mental components are evaluated. The higher the score the better the condition. SF-12 reliability has proven a high internal consistency demonstrating an approximate ICC (Intraclass Correlation Coefficient) of 0.9. [55]

### POMS (profile of mood states)

The Spanish version of POMS [56], with 44 items that represented six conceptual dimensions: anger (11 items), fatigue (6 items), vigour (5 items), friendship (6 items), stress (7 items) and depressed state (9 items). As usual, the answer format consisted of a 5 ordered category response, assigned with values 0 (nothing) to 4 (a lot). All the items were put forward in the same trend except for the item 29-relaxed (corresponding to the state of stress) scores, that were inverted after the test.

### Inter-frailty

The auto-administrated survey based in 10 dichotomous questions in order to detect elder frail people will be carried out [57].

### MMSE (mini-mental state examination) – MEC-30 (mini cognitive examination)

The MEC consists of 30 items grouped into 11 sections, the clinician could carry out in 5–15 min based on the following indications; note that those responsible for carrying out this instrument, both in its original version and its Spanish adaptation [58], warn that the professional should stick to these criteria as far as possible, ensuring maximum objectivity in registering the subject’s responses. The eleven sections are: Orientation to time, Orientation to space, Registration, Attention and Calculation, Memory, Nomination, Repetition (Recall), Comprehension, Reading, Writing and Drawing.

### Picture completion test –WAIS III: performance IQ (perception organization index)

This test consists of displaying to the individuals various drawings that lack an important part, which consequently must be named or pointed out within a 20 s time limit [59] The task ends when 5 consecutive faults are committed. The subject is given 1 point per correctly answered item within the time limit. Among the functions involved in this task we distinguish: concentration; perceptual organization; ability to distinguish essential details of the secondary ones; visual recognition of familiar objects; logical reasoning and visual memory. High scores imply: good perception and concentration; good attention to details; ability to carry out and ability to discriminate relevant from irrelevant details. On the other hand, low scores suggest possible anxiety that could affect concentration and concern for unimportant details.

### Motor skill memory test

Motor skill memory and body awareness will be measured directing a test that consists of performing ten static positions. The examiner executes 10 positions holding each one of them for 10 s. Immediately after, the patient imitates the position in the opposite way, analogous to a mirror. The test ends when the 10 positions have been carried out. This test is included in the “Memory in movement” program, used as a cognitive intervention tool [60].

### Spatial orientation dynamic test-revised

The SODT-R (Spatial Orientation Dynamic Test-Revised) [61] task is to direct two moving objects to a given destination. There are two buttons for each of the moving objects in order to control their course. Pressing the buttons changes the object’s course in one direction, reducing or increasing the angular discrepancy between the current course compared to the ideal course that would stand for 0 deviation.

### Hand – grip test

Hand grip strength test will examine the peak of strength and fatigue resistance. The test is performed with the participant in a sitting position and 90 degrees elbow flexion, neutral pronosupination and flexion, extension or inclination of the wrist will not be allowed. The patient will be asked to hand grip, which will be evaluated with a hydraulic dynamometer JAMAR [62].

### Expanded timed up and go

Expanded TUG is a test to assess mobility, balance and risk of falls in the elderly [63]. The test consists of timing how long the seated patient takes to get up, walk ten metres, return and sit back again on the chair.

### Sample size

To achieve a 80% statistical power to detect differences in the statistical hypothesis testing of the null hypothesis H through a bilateral chi-square test for two independent samples, considering that the significance level is 5%, and based on the studies contained in the Consensus Document on Prevention of Frailty and Falls in the Elderly by the Inter-Territorial Council 2014, a priori it is calculated, based on the outcome variable INTERFRAILTY Q included in one of the trials of such literature, by two means and adding a 15% of the losses (it will be necessary to include 100 subjects in the control group- standard medical practice- and 100 subjects in the experimental group – multimodal intervention program) amounting 200 subjects in the study.

### Data collection

**Fig. 1:**
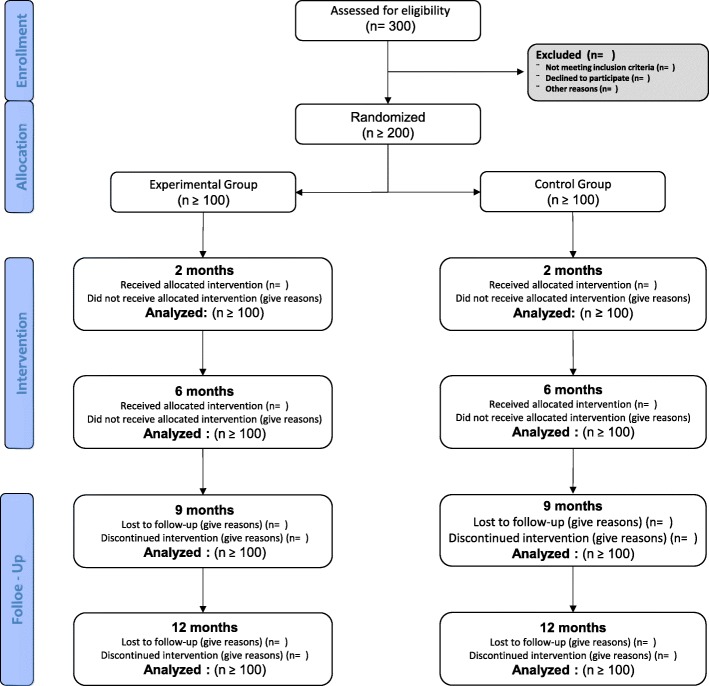
Flow Diagram is presented from the recruitment to the analysis after the follow-up (12 months)

The selection of the population that will take part in the study will be chosen through systematic random sampling. The total population consists of the users that belong to the Clinical Management Unit “Tiro de Pichón” medical lists, identified as frail by the nurse’s assessment and that meet the inclusion criteria as described above. The total population will be sorted by alphabetical order. Having determined the sample size, the sampling interval “k” (the skip) will be calculated using the following formula: k = N/n, where “N” is the total population size and “n” is the sample size. We will establish a random number “a” ranging from 1 to “k” that will be the first subject of the study. The remaining sample will be formed by the following elements: a, a + h, a + 2 h, a + 3 h, until the completion of the required sample size (200 subjects) as detailed further. If one of the selected elements wouldn’t be able to take part of the study, the next following element in the initial alphabetically ordered total population listing will be chosen. Figure 1 shows the Flow Diagram is presented from the recruitment to the analysis after the follow-up (12 months).

Participants will be handed in a patient handout, in which the study’s course will be detailed, and they will be handed in an informed consent, where it will be stated that their enrolment is absolutely voluntary, hence being possible to leave the study when deemed appropriate. Data protection of the participants will be conducted under the following Law: “Ley Orgánica de Protección de Datos de Carácter Personal 19/55”.

### Control group

The participants in this group will undergo an expository briefing on the importance of performing the exercises and handing in the training schedule in order to strengthen the muscle mass, improve the endurance and reskill balance through working out for 6 months. In addition, a medication review will be taken place and they will be handed in a diet adjustment plan. The data collection along time are the same for both control and experimental groups.

### Experimental group

Each one of this group’s participants will undergo the following schedule three times a week every alternative day. It will consist in one specific strength training and another for endurance (sequenced in this order). Before beginning the strength training the participants will carry out two habituating sets to practice the training tests to be performed. After the second habituation session, the 1st RM (maximum repetitions) will be calculated. Each one of them will perform nine exercises: bench press, inclined leg press, seated row, leg extension, back opening, hamstring curl, triceps curl, biceps curl and crunches. These exercises have been chosen to emphasise the synergy between major and smaller muscle groups, as recommended by the American College of Sports Medicine. During the first four weeks, the subjects will perform two series of 18–20 maximum repetitions (RM), shifting to 15–17 RM (weeks 5–9). In weeks 10 to 14, the subjects will perform two series of 15 to 19 RM, shifting to three series of 8–10 RM (weeks 13–16), changing to 6–8 RM (weeks 20–24). The workload per session will be adjusted if the number of repetitions were to be more or less than what was just stated. The recovery time between sets ranged from 90s to 120 s. The estimated time of each strength training session will be 40 min.

An endurance training session will be performed using a cycloergometre and with an intensity relative to the Heart Rate (IRHR). During the first four weeks, the subjects will pedal for 20 min at 80% of their IRHR, moving ahead to 25 min at 85–90% of their IRHR from weeks five to nine. From weeks ten to eighteen, the subjects will cycle for 30 min at 95% of their IRHR and during the last five weeks of training (weeks 20 to 24), the subjects will perform six (6) series of four minutes at 100% of their IRHR, with one minute of active recovery between series.

All training sessions will be carefully supervised by a physiotherapist experienced in therapeutic training. It will be the physiotherapist as well who will conduct the health education during the training sessions. Furthermore, each one of the experimental group patients will undergo a medication review and they will be handed in a diet adjustment plan.

There will be three moments of data collection throughout the intervention: before the intervention, 2 months after the onset and after 6 months. Moreover, there will be a follow-up with two additional measurements: one 3 months and another one 6 months, after the conclusion of the intervention (9 and 12 months from the onset of the intervention, respectively).

The medication review will be focused on the regular, or at least every six months, review of the polypharmacy (intake of more than 5 drugs) insisting on the psychotropic medication due to its greater association to falls and the drugs that can negatively affect people with risk of falling under the STOPP/STAR criteria.

Diet adjustment includes the examination of the variety of nutrients and the protein intake as a protective factor. Anthropometric measurements, weight/height and BMI will be used.

The intervention on the potential hazards at home include conducting the home risk assessment template provided as the Annex 8 of the Consensus document on prevention of frailty and falls in the elderly, and intervening on modifiable elements, obstacles on the floor, mats, cables, lighting type at home, arrangement of the kitchen utensils, bathroom features, type of footwear, clothing and presence of pets.

Team members will reinforce the standard practice by the elements described above in order to improve the condition of frailty.

### Statistical analysis

A descriptive analysis of the socio-demographic variables of the participants will be conducted.

In order to analyse the existent differences in the variable values of interest between the control group and the experimental group, Student’s T-Test will be applied for two independent samples in the case that the normality condition could be accepted, which will be verified by the Saphiro-Wilk test. In case if normality couldn’t be accepted, the appropriate nonparametric test of Mann-Whitey will be conducted.

To analyse the existent differences in the response variables prior to and after the intervention, Student’s T-Test will be carried out for paired samples in the case that the condition of normality could be accepted. In any other case, the appropriate nonparametric test of Wilcoxon will be conducted.

### Difficulties and limitations of the study

The main difficulty of the study is the possibility that the development of the workout sessions could be performed by a clinical physiotherapist specialized in therapeutic training with an ad hoc contract. As an alternative, the researchers themselves would have to acquire the necessary competences to carry out the workout sessions in coordination with the reference Rehabilitation Team (Fisiotherapy Unit – Clinical Management Unit of “Huelin”, Health District of Málaga).

## Discussion

If the hypothesis is accepted, it should achieve further improvements of the neuromuscular and physiological state in the elderly identified as frail, front the standard medical practice. The interaction of various intervention strategies, wherein a supervised workout program, medication review, diet adjustment and health education are included, will achieve further improvements in the elderly identified as frail. This protocol, if beneficial, could be implemented within public health, after certain requirements are resolved with regard to workout program, medication review, diet adjustment and health education, in order to improve the quality if life and the overall health in frail older adults.

### Trial status

This study is open for participant recruitment at the time of submission (enrolling by invitation as of day 1/1/2019).

## Data Availability

Not applicable as this is a study protocol with no gathered data.

## Abbreviations

BMI: Body Mass Index
CONSORT: Consolidated Standards of Reporting Trials
IRHR: Intensity Relative to the Heart Rate
RM: Maximum Repetitions
SPPB: Short Physical Perfomance Battery
